# The potential role of kelp forests on iodine speciation in coastal seawater

**DOI:** 10.1371/journal.pone.0180755

**Published:** 2017-08-11

**Authors:** Jennifer Gonzales, Teresa Tymon, Frithjof C. Küpper, Matthew S. Edwards, Carl J. Carrano

**Affiliations:** 1 Department of Chemistry and Biochemistry, San Diego State University, San Diego, California, United States of America; 2 Oceanlab, University of Aberdeen, Newburgh, Scotland, United Kingdom; 3 Department of Biology, San Diego State University, San Diego, California, United States of America; SPAIN

## Abstract

Kelps have a major role in marine and atmospheric iodine cycling in the coastal zone of temperate regions, with potential wide-ranging impacts on ozone destruction in the coastal marine boundary layer. However, little is known about the impact of kelp forests on iodine speciation in coastal sea water. To address this, we examined iodide and iodate concentrations in seawater in and around a giant kelp forest near San Diego, CA, USA, and a nearby site that was not influenced by kelp biology. Our data shows that while both iodide and iodate concentrations remained unchanged during the year at the nearby site, these concentrations changed significantly in and around the kelp forest, and were strongly related to changes in kelp canopy biomass. In particular, iodide reached its highest concentration and iodate reached its lowest concentration during the summer when the kelp canopies were near their maximum, while the opposite pattern was observed during the winter and spring when the kelp canopies were near their minimum. Further, comparisons of these changes with corresponding changes in seawater temperature and wind speed indicated that these relationships were relatively small compared to those with changes in kelp biomass. Together, our data show a strong relationship between kelp biomass and iodine metabolism.

## Introduction

Marine production of organic halogenated compounds has been proposed as an important link between ocean biology, atmospheric composition, and climate [1–5]. In particular, iodine atoms released from the photooxidation of organic and inorganic halogenated compounds enter catalytic ozone destruction cycles and can indirectly lead to a reduction in tropospheric ozone production by suppressing levels of nitrogen oxides [6]. The climate relevance of the link between ocean biology and atmospheric halogens (including iodine) was demonstrated by estimating the change in the ozone radiative forcing due to halogen-driven ozone loss using a climate model [7,8]. These halogenated compounds enter the stratosphere through rapid convection from the marine boundary layer (MBL) [9], and once there may be more efficient than chlorine in destroying stratospheric ozone, a key greenhouse gas and air pollutant [10]. Recent models have shown that iodine enters the stratosphere at larger concentrations than currently assumed by WMO [11]. For example, computer simulations suggest tropospheric ozone may be reduced by 5–30% compared to simulations without halogenated compounds [5,12] and reactive iodine, together with bromine, may be responsible for up to 50% of ozone destruction in the MBL [13]. Such ozone depletion may contribute approximately −0.10 W m^−2^ to the radiative flux at the tropical tropopause, which is of similar magnitude to the contribution of tropospheric ozone to the present-day radiative balance [14]. Iodine compounds can also impact climate through modification of nitrogen oxide (NOx) and hydrogen oxide (HOx) cycles, and through the oxidation of dimethyl sulfide (DMS) [4], with resulting effects on the lifetimes of other climatically important trace gases [15,16]. Up to date, only two global models have evaluated the role of atmospheric iodine chemistry in the global concentration of atmospheric oxidants [17,18].

In addition to its widespread, potentially global, effects on climate, reactive iodine may impact local to regional patterns of cloud formation over coastlines that are rich in kelp forests via nucleation and growth of particles involving iodine oxides [19–21]. Although early work identified di-iodomethane (CH_2_I_2_) as the likely precursor to iodine oxide particles (IOPs) [19], it is now clear that molecular iodine (I_2_) efflux from seaweed dominates over organic iodine emissions by perhaps three to five orders of magnitude [20,22,23]. Because I_2_ is very short-lived, the extent of IOP influence is likely to be restricted to the coastal zone where kelp forests occur [24], but recent data demonstrate the ability of such particles to grow to condensation nuclei [25] and thus have at least regional meteorological radiative effects. However, there is surprisingly little knowledge about the function of kelp forests in global biogeochemical cycles, with the exception of some recent interest in the aftermath of the Fukushima nuclear disaster. For instance, while we know little about halogen metabolism in giant kelp, *Macrocystis pyrifera*, the species appears to exhibit iodide uptake rates in the range of 8 x 10^−3^ fmol cm^-2^ min^-1^ [26], relatively low tissue concentrations of around 0.48 mg g^-1^ [27], and significant methyl iodide emissions [28–30]. Further, even less is known about marine-atmospheric halogen transfers in regions where *Macrocystis* forms extensive forests along coastal rocky reefs; in particular, the west coasts of North and South America, South Australia, Africa, and the Sub-Antarctic Islands. This seems surprising considering that a related North Atlantic kelp species, *Laminaria digitata*, is known to be a major biogeochemical pump of iodine from the ocean to the atmosphere and the strongest iodine accumulator currently known among all living systems [1,21,31,32]. However, the thallus structure of *Laminaria* is quite different from *Macrocystis*. Specifically, while *Laminaria* occurs below the surface and becomes exposed to the atmosphere only intermittently (e.g. during spring low tide events), a large portion of the *Macrocystis* thallus floats on the sea surface and is thus continuously exposed to the atmosphere. Given that the periodic exposure of *Laminaria* to atmospheric ozone combined with high irradiance results in particle bursts in MBL [19,21,23], there are a number of obvious implications for *Macrocystis*.

Since seawater is the ultimate source of iodine and its oxidation products [1], its concentration and speciation are important parameters. The distribution of iodine in the surface ocean has recently been reviewed by Chance et al. [33], revealing that the total iodine concentration in seawater is generally constant at about 450–550 nM. This iodine occurs in two main forms; iodate (IO_3_^-^) and iodide (I^-^), plus variable small quantities of organoiodine species. Of these, iodate is considered the thermodynamically stable form under oxygenated seawater conditions and should be the primary iodine-containing species in the water column. Indeed, iodate exhibits median concentrations of approximately 423 nM while iodide exhibits median concentrations of approximately 77 nM [34]. The oxidation of iodide back to iodate, although thermodynamically favourable, is a six-electron process that is complicated by the formation of molecular iodine, which under current average pH of seawater (8.1) exists as HOI and provides reactive iodine as I^+^ to organic matter [31]. Thus, once reduced, the kinetics of reoxidation of iodide back to iodate are extremely slow, thereby allowing the build-up of iodide in the seawater [35]. This build-up is particularly prevalent near the sea surface, where it constitutes up to 50% of total dissolved iodine in the euphotic zone, but decreases with depth, approaching 1 nM in deep waters. In contrast, the concentration of iodate in the ocean also varies with depth, but is generally lower at the surface and increases with depth [36]. Also, iodide concentrations are significantly related to latitude such that they exhibit a pronounced increase between 20 and 50°, with concentrations averaging more than 100 nM in the lower latitudes but less than 50 nM in the higher latitudes, although there are major exceptions [33].

The fact that iodide concentrations follow a depth dependence corresponding to the euphotic zone and display an anti-nutrient profile strongly suggests that biological activity is the source of the reduced iodine in the oceans [36–40]. However, the nature and source of iodate reduction activity remain controversial despite an extensive literature covering macroalgae, phytoplankton and bacteria, all having been suggested to be involved along with a number of abiotic processes [34, 35, 41–43]. In oxygenated waters, for example, phytoplankton have been suggested to reduce iodate to iodide as a side reaction of nitrate reductase [44]. However, *in vitro* experiments with nitrate reductase indicate that this may not be a major process, but rather it may depend on phytoplankton community composition, with different taxa having differing abilities to reduce iodate [45]. Another more recent hypothesis put forward to explain the many disparate past observations suggests that the transformation from iodate to iodide is driven by cell senescence of marine phytoplankton [43]. Here reduction of iodate occurs not through active cellular metabolism of iodate but rather via leakage of sulphur-containing reducing agents, i.e. glutathione, cysteine etc. accompanying phytoplankton cell senescence. Regardless of the particular processes involved, a number of studies have found that the iodate concentrations diminish while those of iodide increase as the coast is approached, suggesting that coastal waters are particularly effective points of iodate reduction [33]. Since many coastal ecosystems are dominated by macroalgae that are known to have highly evolved halogen metabolisms and high biological productivity rates, it is reasonable to suppose that these organisms could potentially influence iodine speciation in the coastal zone, especially if they occur in high abundance. In particular, *Macrocystis*, as one of the largest living organisms on the planet with a high growth rate of up to 30 cm/day is widely considered an ecosystem engineer due to its ability to form dense forests that create biogenic structure, enhance biodiversity, and modify hydrodynamic, light and nutrient conditions within the forest boundaries [46–48]. Consequently, changes in the distribution and/or abundance of *Macrocystis* forests can have dramatic impacts on energy and carbon flow and nutrient cycling in the coastal zone, as has been observed for terrestrial forests [49]. Several possibilities could be hypothesized for how *Macrocystis* could affect iodide concentrations in and around coastal kelp forests. For example, it is known that *Macrocystis* takes up iodide from seawater in an iodoperoxidase-dependent process, which could be a possible mechanism for the removal of iodide from seawater [50]. Conversely, the known efflux of iodide from this species under stressful conditions represents a possible iodide output [50] that is comparable to other macroalgae [21, 41, 51]. This makes *Macrocystis* an excellent model system with which to study how, or if, biological activity affects iodine speciation [27, 50]. In particular, changes in iodide concentrations within and around *Macrocystis* forests are expected, and the nature of the effects (i.e. increased or decreased iodide) may inform us about how or if iodide is biologically processed by the species. Determining the factors that affect iodine speciation is critically important for our understanding of the global biogeochemical cycle of iodine. Thus, this study was conducted in order to assess the influence of *Macrocystis* kelp forests on inorganic iodine speciation in the vicinity of Point Loma, San Diego (California, USA).

## Materials and methods

### Water sampling

To determine variability in seawater iodide concentrations within and around the Point Loma kelp forest (32°43’N, 117°16’W) in Southern California, USA, seawater samples were collected at 0 and 10 m depths from five locations within and/or adjacent to the forest several times (generally monthly) each season from August 2014 to July 2015 (hereafter, Winter = December 2014 through February 2015, Spring = March through May 2015, Summer = August and September 2014 and June and July 2015). No permits were required. These locations were designated as: nearshore, inside of forest, mid forest, immediately outside of forest, and 500 m outside of the forest, as determined visually on site (S1 Fig). The seawater was collected using Niskin bottles and the samples immediately transferred to acid-washed polyethylene bottles for analysis. All seawater samples were filtered through a 0.45 μM Millipore membrane to remove any biological components, and it was determined beforehand that such filtration has a negligible effect on measured iodide and total reducible iodine concentrations. Iodate concentrations were also determined for these samples for the period January–July 2015. In addition, surface seawater samples were collected from the Scripps Pier, La Jolla (32°53’N, 117°15’W), an area approximately 10 nautical miles north of Pt. Loma which is devoid of *Macrocystis*, on a monthly basis from January to July 2015 (a period that encompassed the iodate analysis), and used as a baseline for iodide and iodate concentrations not being locally influenced by kelp biology. Water temperature and wind speed/direction data were obtained from the Scripps Pier data site, http://www.sccoos.org/data/Piers/.

### Iodide measurements

Iodide concentrations were determined by cathodic stripping square wave voltammetry (CSSWV) according to Luther et al. [37]. Briefly, 1.3 mL of sample was pipetted to a voltammetric cell to which Na_2_SO_3_ (1M) and Triton X-100 (0.4% v/v) were added to increase the sensitivity for the iodide peak. The sample was purged for several minutes with nitrogen to remove oxygen. System parameters were as follows: deposition E (0 mV), deposition t (30 s), initial potential (-100 mV), final potential (-500 mV), step E (2 mV), SW Amp (20 mV), SW frequency (100 Hz). Analysis was performed on a BASi controlled growth mercury electrode model MF-9058 interfaced with Epsilon software. A platinum wire served as the auxiliary electrode and an Ag/AgCl electrode was used as the reference electrode. The optimum linear range for the media used here produced a signal up to 0.2 μA; appropriate sample dilutions were performed using MQ H_2_O. Iodide concentrations were determined (in triplicate) by the method of standard additions using known concentrations of potassium iodide. Total reducible iodine (TRI) concentrations were determined in duplicate samples by reducing all of the iodine in the seawater samples into iodide using an ascorbic acid solution as previously described [52]. Samples were allowed to temperature equilibrate for 24 hours prior to electrochemical analysis. Iodate was determined by taking the difference between total reducible iodine and free iodide in samples as concentrations of non-reducible organoiodine species are expected to be very low.

### Kelp canopy estimates

Estimates of kelp canopy biomass for the Point Loma kelp forest during 2014 and 2015 were provided by T. Bell from the UCSB Long-term Ecological Research Program. Briefly, canopy coverage was estimated using Landsat imagery and processed using image analysis with the program ENVI, and converted to biomass, in wet kg, using known relationships between canopy area and biomass.

### Statistical analysis

All analyses were done using SYSTAT Ver. 12. Prior to testing, all data were inspected to ensure they met the assumptions of normality and homoscedasticity, and any data not meeting these assumptions were transformed and retested to ensure the problems were corrected. Changes in iodide and iodate concentrations observed at the Scripps Pier were each evaluated as a function of sample month using separate linear regressions, with Julian month as the independent variable and either iodide or iodate concentration as the dependent variable. The relative amount of temporal variation in each of these measures was then compared between species by calculating their coefficient of variations (CVs). Following this, variability in iodide and iodate concentrations among sample locations relative to the Point Loma kelp forest, depths and seasons were each evaluated using separate three-way Model I ANOVAs. The relative amount (%) of variation accounted for by each factor and their interactions were determined by calculating their magnitude of effects (ω^2^) using the Variance Components analysis procedure in SYSTAT and interpreted according to Graham and Edwards [53]. For iodide, pairwise differences among sample seasons were assessed for each depth using Tukey’s HSD post hoc tests on the significant season*depth interaction (see Results). Following this, specific hypotheses concerning variation in iodide and iodate concentrations among sample locations were assessed within season separately using separate one-way ANOVAs as *a prior* defined post hoc tests. Also, following identification of a significant season effect for iodate (see Results), variation in iodate concentrations among pairs of sample seasons was evaluated using Tukey’s HSD post hoc tests. Finally, the influences of seawater temperature and wind speed on both iodide and iodate concentrations were evaluated across seasons and depths for each species using separate three-way Analyses of Covariance, with location as a categorical factor, and wind speed and water temperature as covariates. Following the identification of significant overall effects of water temperature on both iodide and iodate concentrations (see Results), the functional relationships between water temperature and both iodide and iodate concentrations were evaluated using separate linear regressions. Also, following the identification of a significant Location X Wind speed interaction for iodide (See Results), the functional relationship between wind speed and iodide concentration was evaluated for each location separately using linear regressions.

## Results

### Scripps Pier

**Fig 1:**
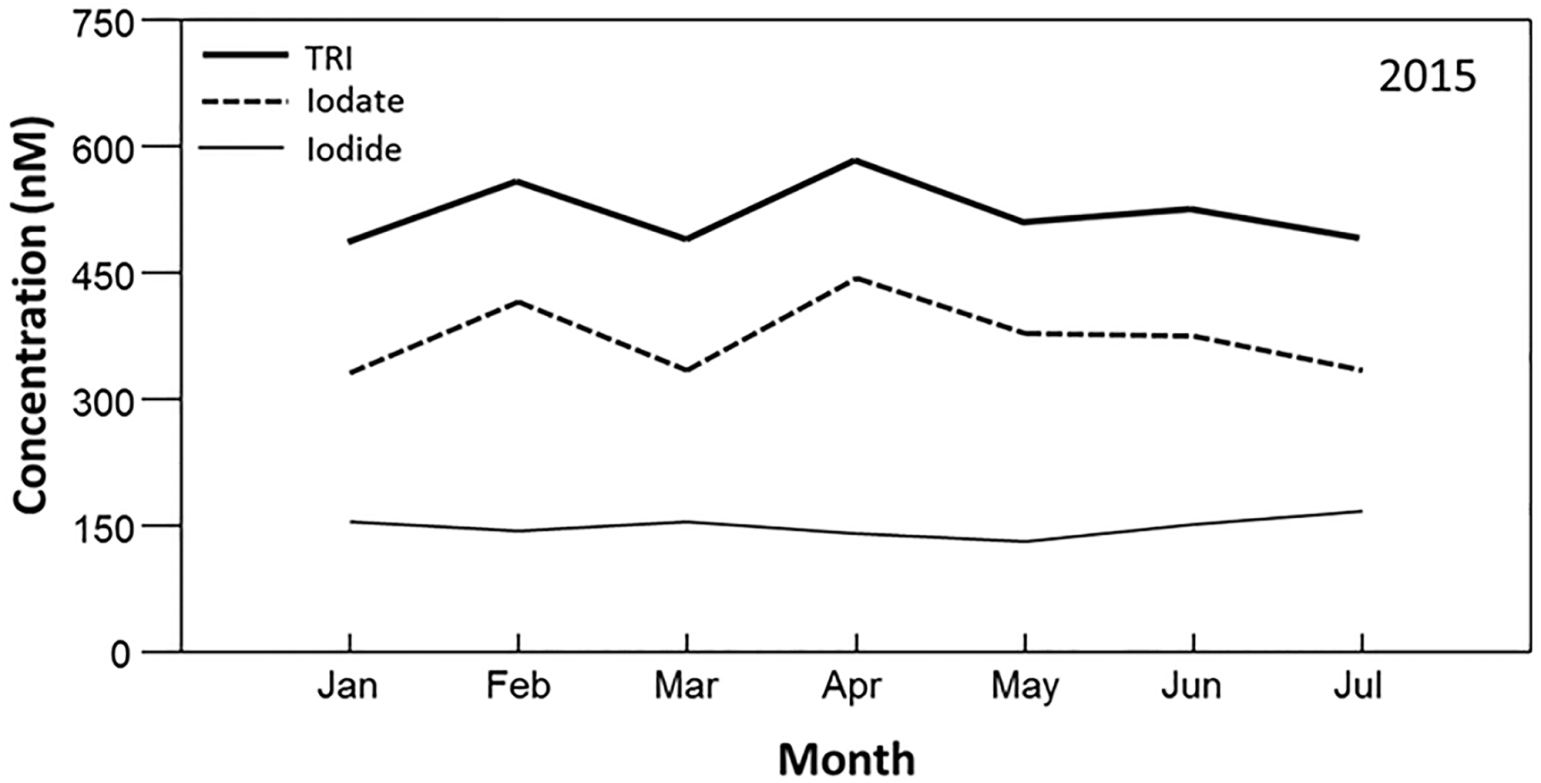
Mean concentrations of iodide, total reducible iodine and iodate (by difference) in surface waters at the Scripps Pier, La Jolla, CA between January and July 2015.

Monitoring seawater total reducible iodine (TRI), iodide and iodate concentrations at Scripps Pier monthly from January through July 2015 revealed that the concentrations of these species were essentially unchanged throughout the monitoring period (Regressions: iodide, F_1,5_ = 0.195, p = 0.677; iodate, F_1,5_ = 0.014, p = 0.908). Specifically, the concentration of TRI was, on average, 523 ± 36 nM (mean ± sd), the concentration of iodide was, on average, 150 ± 11 nM, and iodate was, on average, 373 ± 43 nM (Fig 1). These values are in accord with other measurements taken in low latitude coastal waters [33]. However, although these values were statistically unchanged as a function of sample month, there was slightly more relative temporal variation in iodate concentration (CV = 0.11) than iodide concentration (CV = 0.08) or TRI (CV = 0.07), and the temporal variation observed in TRI appeared more closely related to variation in iodate than iodide (Fig 1).

### Pt. Loma kelp beds

**Fig 2:**
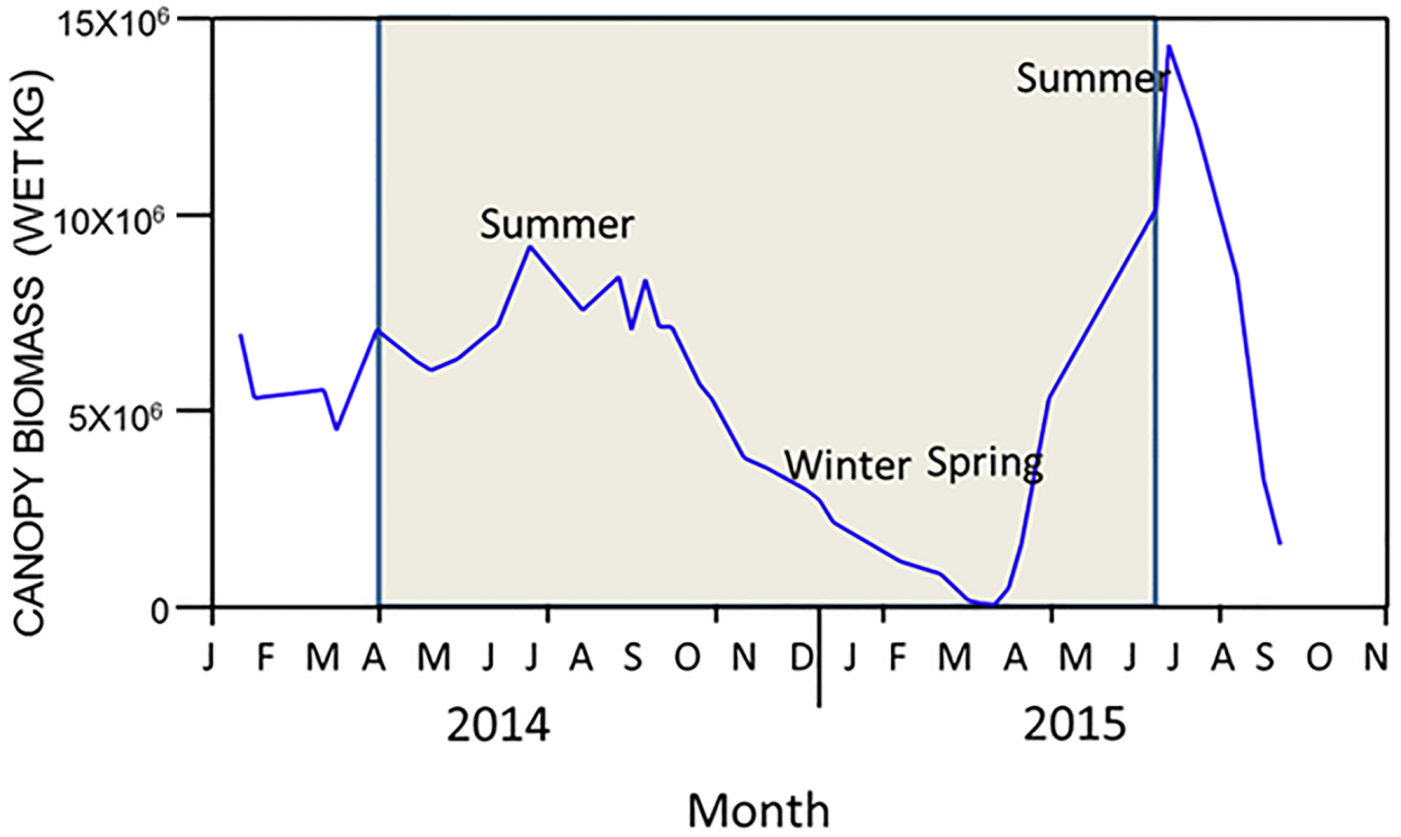
Pooled iodide concentrations (mean ± standard deviation) as a function of season at five locations within the Pt. Loma kelp forest. The dotted line represents the mean iodide concentration at the Scripps Pier control site.

**Fig 3:**
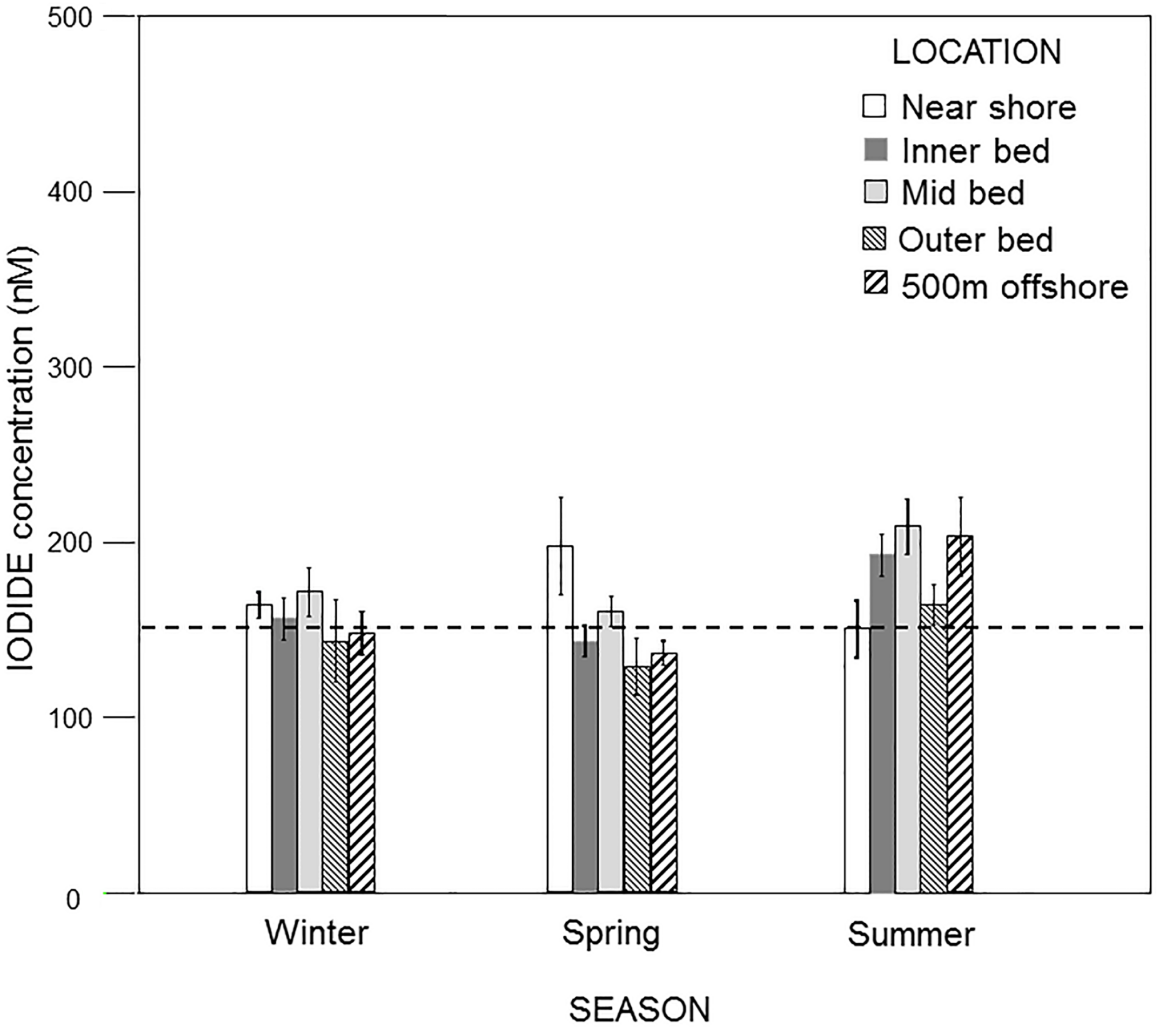
Kelp forest canopy biomass from 2014–2015 as estimated from Landsat imagery, and expressed as biomass in wet kg. The grey box represents the time frame of this study.

In contrast to the Scripps Pier site, iodide and iodate concentrations in and around the Point Loma kelp forest both varied considerably over the course of the study. Indeed, while iodide concentrations in and around the forest were generally higher (166 ± 58 nM) than the concentrations at the Scripps Pier when averaged across all locations, depths and seasons, they varied significantly among locations relative to the kelp forest (ANOVA: F_4,188_ = 2.58, p = 0.039) but not between sample depths (F_1,188_ = 2.303, p = 0.131) (Table 1). Specifically, iodide values were generally lowest and most similar to the Scripps Pier values at the "outer edge" of the forest (149 ± 51 nM) and "500 m outside" of the forest (164 ± 67 nM), and highest and most dissimilar to the Scripps values in the “middle” of the forest (181 ± 56 nM). However, these concentrations varied significantly among seasons (F_2,188_ = 15.03, p < 0.001), which accounted for approximately 15% of the total variation observed, but the relative differences among locations remained consistent (Location x Season interaction: F_8,188_ = 0.310, p = 0.962) (Table 1). In particular, when averaged across locations in and around the kelp forest, iodide concentrations were generally greatest and most dissimilar to the Scripps values during the summer (178 ± 56 vs. 160 ± 11 nM, respectively) when kelp canopy biomass was near its maximum. This was especially evident in the “middle” of the forest where iodide concentrations reached values as high as 209 ± 60 nM (Table 1, Fig 2). In contrast iodide concentrations were generally lowest and most similar to the Scripps values in the winter (157 ± 49 vs. 150 ± 8 nM, respectively) and spring (144 ± 35 vs. 143 ± 12 nM, respectively) when the kelp canopy biomass was near its minimum (Figs 2 and 3). Interestingly, iodide concentrations did not differ among locations when examined within any one season separately (ANOVAs: winter, F_4,76_ = 0.78, p = 0.555; spring, F_4,49_ = 1.41, p = 0.244; summer, F_4,78_ = 1.06, p = 0.379). Further, while iodide concentrations again did not differ between sample depths, the differences observed among seasons did vary significantly depending on the sample depth at which they were considered (Season X Depth interaction, F_2,188_ = 4.22, p = 0.016), with this interaction accounting for approximately 29% of the total variation observed (Table 1). Indeed, iodide concentrations were significantly lower in the spring than in summer at both the surface and 10 m depth (Tukey’s: p = 0.001 and 0.006, respectively), but they did not differ between the spring and winter at either depth (p = 0.208 and 0.994, respectively). In contrast, iodide concentrations were significantly greater in the summer than in the winter at the surface (p = 0.002) but they did not differ at 10 m (p = 0.231). Likewise, iodide concentrations were significantly greater at the surface than at 10 m depth in summer (Tukey’s: p = 0.029), but they did not differ between the two depths in either the spring (p = 0.781) or winter (p = 0.669).

**Table 1:** Results of a 3-way Model I analysis of variance testing variation in iodide concentrations among sample locations relative to the Point Loma kelp forest, sample depths (surface and 10 m) and seasons.

Source	SOS	df	MS	F-ratio	p-value	w2
LOCATION	0.031	4	0.008	2.581	**0.039**	0.023
DEPTH	0.007	1	0.007	2.303	0.131	0.012
SEASON	0.091	2	0.045	15.035	**0.001**	0.149
DEPTH*LOCATION	0.004	4	0.001	0.327	0.860	0.000
SEASON*LOCATION	0.007	8	0.001	0.310	0.962	0.000
SEASON*DEPTH	0.026	2	0.013	4.227	**0.016**	0.285
SEASON*DEPTH*LOCATION	0.023	8	0.003	0.938	0.486	0.000
Error	0.569	188	0.003			0.530

w^2^ denotes the relative amount (%) of total variation in iodide concentrations that is accounted for by each factor and their interactions.

Bold-faced type indicates significant variation was found for that factor.

**Fig 4:**
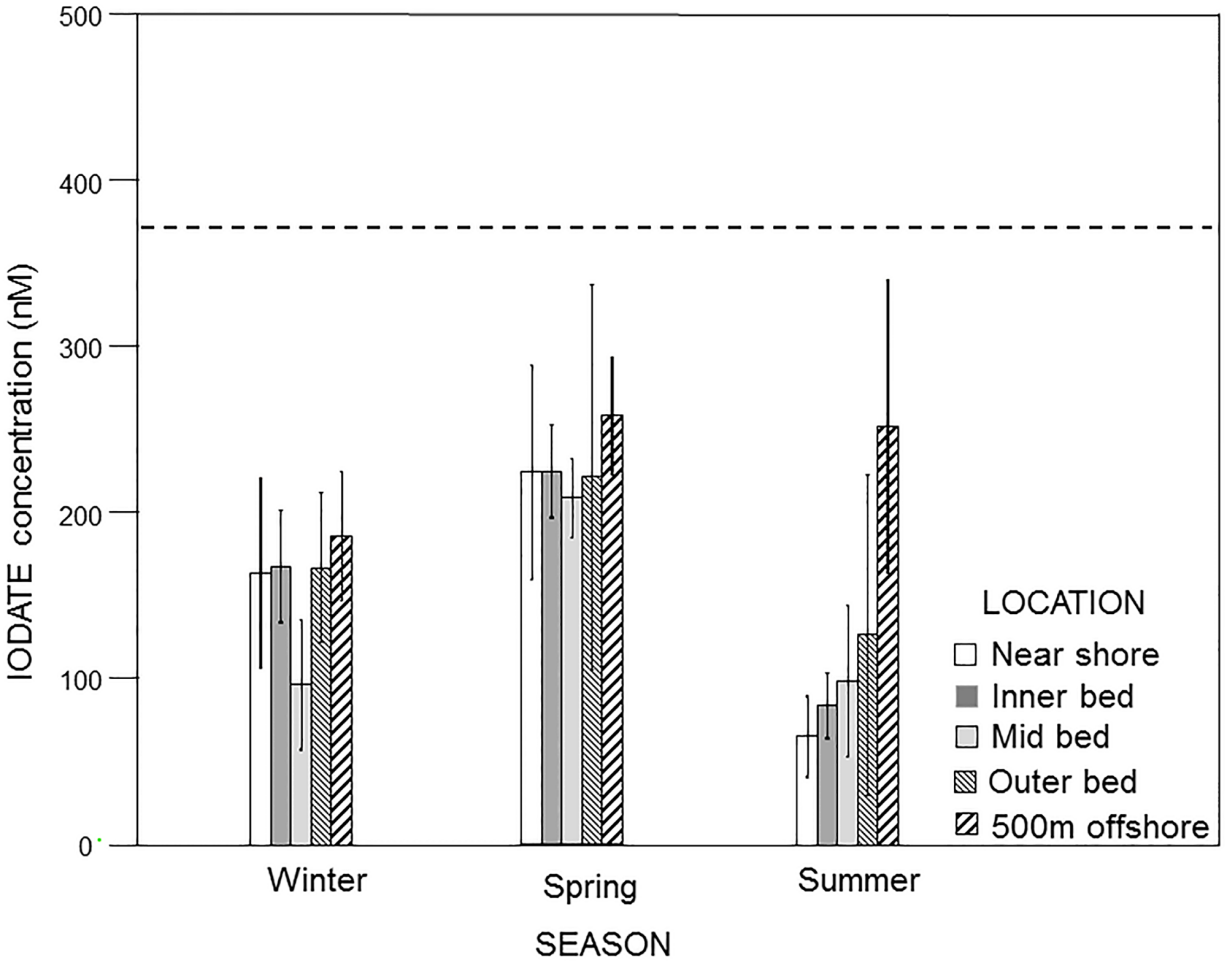
Pooled iodate concentrations (mean ± standard deviation) as a function of season at five locations within the Pt. Loma kelp forest. The dotted line represents the mean iodate concentration at the Scripps Pier control site.

In contrast to iodide, iodate concentrations in and around the Point Loma kelp forests did not differ significantly among locations relative to the Point Loma kelp forest (ANOVA: F_4,26_ = 2.08, p = 0.113) or between sample depths (F_1,26_ = 3.22, p< 0.085), but they did vary significantly among seasons (F_2,26_ = 6.17, p = 0.006), which accounted for approximately 18% of the total variation observed (Table 2, Fig 4). Further, the differences observed among seasons were consistent across sample locations (Location x Season interaction: F_8,26_ = 0.701, p = 0.688) and depths (Season X Depth interaction, F_2,26_ = 1.37, p = 0.272). When averaged across all locations, depths and seasons, iodate concentrations in and around the kelp forest were consistently and dramatically lower than the concentrations at the Scripps Pier (193 ± 114 vs. 373 ± 43 nM, respectively). These, values were highest and most similar to the Scripps Pier values at the "500 m outside" bed position (231 ± 95 nM), and lowest and most dissimilar to the Scripps Pier values in the “middle” of the forest (134 ± 78 nM). This was especially evident in the summer of 2015 when the kelp canopies were near their maximum and iodate values within the middle of the forest fell to as low as 99 ± 76 nM. Further, when averaged across locations in and around the kelp forest, iodate values followed the opposite seasonal trend to iodide, with lower values generally observed in summer of 2015 (126 ± 104 nM) when the canopies were near their maximum and higher values observed during the winter of 2014 (154 ± 69 nM) and spring 2015 (226± 95 nM) when the canopies were near their minimum (Figs 3 and 4). Specifically, iodate concentrations were significantly higher in the spring than in the summer (Tukey’s: p = 0.006) and marginally higher in the spring than the winter (p = 0.060), but they did not differ between the summer and winter (p = 0.636). Further, as with iodide, iodate concentrations did not vary among sample locations in any of the seasons when examined separately (ANOVAs: winter, F_4,13_ = 0.961, p = 0.461; spring, F_4,15_ = 0.12, p = 0.972; summer, F_4,13_ = 2.92, p = 0.063). Also, as with iodide, iodate concentrations appeared generally lower at the near shore station than in the kelp forest or offshore of it in the summer, but they again did not appear to vary among locations in either the winter or spring (Fig 4).

**Table 2:** Results of a 3-way Model I analysis of variance testing variation in iodate concentrations among sample locations relative to the Point Loma kelp forest, sample depths (surface and 10 m) and seasons.

Source	SOS	df	MS	F-ratio	p-value	w2
LOCATION	0.067	4	0.017	2.076	0.113	0.061
DEPTH	0.026	1	0.026	3.217	0.085	0.051
SEASON	0.100	2	0.050	6.176	**0.006**	0.177
DEPTH*LOCATION	0.025	4	0.006	0.785	0.545	0.000
SEASON*LOCATION	0.045	8	0.006	0.701	0.688	0.000
SEASON*DEPTH	0.022	2	0.011	1.369	0.272	0.025
SEASON*DEPTH*LOCATION	0.024	8	0.003	0.375	0.924	0.000
Error	0.210	26	0.008			0.685

w^2^ denotes the relative amount (%) of total variation in iodide concentrations that is accounted for by each factor and their interactions.

Bold-faced type indicates significant variation was found for that factor.

Iodide concentrations in and around the Point Loma kelp forest were significantly related to both wind speed (ANCOVA: F_1,203_ = 33.80, p = 0.001) and water temperature (F_1,203_ = 10.28, p = 0.002), though these factors explained only 1.7% and less than 0.1% of the overall variance in iodide values, respectively (Table 3, S2 Fig). Rather, when considered along with wind speed and water temperature, iodide concentrations were more strongly impacted by their location relative to the forest (F_4,203_ = 3.45, p = 0.009), which explained 40.1% of the overall variance, and by natural variability among samples (i.e. error), which explained 58.1% of the variance in iodide values. Specifically, when considered on their own, iodide concentrations were positively related to water temperatures (i.e. warmer waters were characterized by greater concentrations of iodide), a pattern that was consistent across all sample locations (Location X Water Temperature interaction, F_4,203_ = 1.57, p = 0.181). However, this may be confounded by the fact that the warmer waters were observed during summer when kelp canopies were at their maximum biomass and iodide concentrations in and around the kelp forest were at their maximum, and vice versa in the winter (Figs 1–3). In contrast, the relationship between wind speed and iodide concentration varied among the different locations (Wind X Location interaction, F_4,203_ = 9.60, p = 0.001), though this interaction explained less than 1% of the variance in iodide values (Table 3). In particular, iodide concentrations were positively related to wind speed at the near shore (Regression, F_1,44_ = 67.87, p < 0.001), inner (F_1,44_ = 21.34, p < 0.001), mid (F_1,44_ = 14.54, p < 0.001) and outer (F_1,32_ = 11.31, p = 0.002) locations, but they were not related to wind speed at the 500 m offshore location (F_1,44_ = 0.55, p = 0.46). Together, these data indicate that while significant relationships could be identified between iodide and both wind speed and water temperature, the influences of these factors were very small in comparison to those of location around the forest and simple natural (error) variability (Table 3), and may have been driven more by corresponding seasonal changes in kelp canopy biomass (Table 1, Figs 1–3).

**Table 3:** Results of a 3-way Analysis of Covariance testing variation in iodide concentration among sample locations relative to the Point Loma kelp forest and its relationship with wind speed and water temperature.

Source	SOS	df	MS	F-ratio	p-value	w2
LOCATION	0.032	4	0.008	3.448	**0.009**	0.401
WIND	0.079	1	0.079	33.804	**0.001**	0.017
WATER	0.024	1	0.024	10.278	**0.002**	< 0.001
LOCATION*WIND	0.090	4	0.023	9.602	**0.001**	< 0.001
LOCATION*WATER	0.015	4	0.004	1.578	0.181	< 0.001
Error	0.476	203	0.002			0.581

ω^2^ denotes the relative amount (%) of total variation in iodide concentrations that is accounted for by each factor and their interactions.

Bold-faced type indicates significant variation was found for that factor.

Similar to iodide, iodate concentrations were also significantly related to water temperature (ANCOVA, F_1,41_ = 7.45, p = 0.009), but they were not related to wind speed (F_1,41_ = 0.59, p = 0.446) (Table 4). These factors again each explained only 0.5% and less than 0.1% of the observed variance in iodate values, respectively. Rather, even though iodate concentrations did not vary significantly among locations relative to the kelp forest when considered along with wind speed and water temperature (ANCOVA: F_4,41_ = 1.65, p = 0.178), variability among locations did account for 56.1% of the observed variance in iodate values, and consequently appeared to influence iodate concentrations more strongly than water temperature or wind speed did. Further, similar to iodide, natural variability among samples accounted for 43.2% of the variance in iodate values. Specifically, when considered on their own, iodate concentrations were negatively related to water temperature (i.e. warmer waters were characterized by lower concentrations of iodate) (Regression, F_1,54_ = 6.73, p = 0.012), a pattern that was consistent across all sample locations (ANCOVA: Location X Water Temperature interaction, F_4,41_ = 1.11, p = 0.363). However, as with iodide, this again was likely confounded by the fact that the warmer waters were observed during summer when kelp canopies were at their maximum biomass and iodate concentrations in and around the kelp forest were at their lowest vales, and vice versa in the winter (Figs 1, 3 and 4). Together, these data indicate that while a significant relationship could be identified between iodate concentrations and water temperature, the influence of this relationship was very small in comparison to those of location within the forest and simple natural (error) variability (Table 4), and may again be driven more by corresponding seasonal changes in kelp canopy biomass (Table 2).

**Table 4:** Results of a 3-way Analysis of Covariance testing variation in iodate concentration among sample locations relative to the Point Loma kelp forest and its relationship with wind speed and water temperature.

Source	SOS	df	MS	F-ratio	p-value	w2
LOCATION	0.057	4	0.014	1.658	0.178	0.561
WIND	0.005	1	0.005	0.592	0.446	0.000
WATER	0.064	1	0.064	7.451	**0.009**	0.005
LOCATION*WIND	0.008	4	0.002	0.227	0.922	0.000
LOCATION*WATER	0.038	4	0.010	1.114	0.363	0.001
Error	0.353	41	0.009			0.432

ω^2^ denotes the relative amount (%) of total variation in iodide concentrations that is accounted for by each factor and their interactions.

Bold-faced type indicates significant variation was found for that factor.

## Discussion

The marine biogeochemistry of iodine is likely important to global climate since the marine environment is likely the major source of emission of both molecular iodine and iodinated organic compounds into the atmosphere [1]. There, these compounds can destroy tropospheric ozone, modify nitrogen oxide and hydrogen oxide cycles, and increase cloud formation [2]. Together, these can increase rates of atmospheric warming, especially over coastal areas where iodine cycles are enhanced by biological activity of primary producers. Consequently, one of the original motivations for this work was to test the hypothesis that reduction of iodate, in at least some coastal ecosystems, occurs due to leakage of sulfur containing reducing agents (i.e. glutathione, cysteine, etc.) that accompany marine phytoplankton cell senescence. A natural laboratory for testing this hypothesis would make use of intense near shore harmful algal blooms (HABs). The Scripps Institution of Oceanography (SIO) Pier is a long-term HAB coastal monitoring site in the Southern California Bight that is well known to experience large cycles in phytoplankton abundance, such as the one that occurred during October 2011 [54]. During these blooms, phytoplankton cell numbers can increase from just a few to more than 150,000,000 cells L^-1^. We proposed to monitor iodine speciation pre-bloom, during the bloom maximum, and post-bloom at this location with the expectation that detectable increases in iodide concentrations post-bloom would be consistent with the cell senescence model. However contrary to expectations, there were no phytoplankton blooms at Scripps Pier during any part of our 2014–2015 field campaign. Indeed we found that by examining the SIO-Scripps Pier HAB monitoring report monthly, total phytoplankton abundance was unusually low and unchanged during the year, possibly due to low nutrient availability that resulted from the very strong 2014–2015 El Niño Southern Oscillation. Consequently, the Scripps Pier site became not a testing site for the influence of phytoplankton activity on iodine speciation, but rather a near ideal "non-biological" control site where values were consistent with many other seawater iodine speciation studies [33] and apparently unaffected by changes in water temperature and/or wind speed.

In contrast to observations at the Scripps Pier site, iodide and iodate concentrations in and around the Pt. Loma kelp *Macrocystis* forest exhibited significant, and in some cases dramatic, changes during the year that varied according to the location relative to the forest, water temperature, wind speed, and especially seasonal changes in kelp abundance. Specifically, in the case of iodide, values in and around the forest were either similar to, or slightly lower than, those observed at the Scripps Pier site during the winter of 2014 and spring of 2015, when kelp was in low abundance. However, in the summers of 2014 and 2015, when kelp abundance was high, iodide concentrations in and around the kelp forest were generally greater than at the Scripps Pier site. This, along with changes in water temperature and wind speed could have caused increased photooxidative and temperature stress, resulting in efflux of iodide into surrounding seawater for antioxidant purposes [21, 51, 55], provided that the kelp has a sufficient iodide reservoir for sustaining such an efflux over a period of months. However, the most surprising observation from this study was that while seawater iodide concentrations increased only slightly in and around the kelp forest in the summer, iodate decreased dramatically compared to the Scripps Pier site. The observation that iodate decreased far more than iodide increased likely means that not only is iodine speciation being affected in and around kelp forests, but also that a considerable amount of the iodate is removed from the water column rather than simply being reduced to iodide. The simplest hypothesis to account for this observation is that iodate is being actively taken up by *Macrocystis* or its associated kelp forest species. This is an unexpected result in that previous work on the stipitate kelp *Laminaria digitata* in the eastern North Atlantic showed that iodide was the primary form of iodine that is taken up by the kelp and stored in areas of the thallus, as such in the apoplast [32]. In contrast, this study, coupled with recent work by Tymon et al. [50], suggests that there may be fundamental differences in the halogen metabolisms among the various kelp species. However, a number of alternative theories to account for the reduction in both iodate and TRI during the summer months exist. For example there could be as yet unknown abiological reactions of dissolved organic matter (DOM) with iodate leading to undetectable products or the existence of large amounts of organoiodine species in the seawater around the kelp beds which are undetectable in our assay and have been assumed to be small. However, even in these cases *Macrocystis* is the only likely source of either reactive DOM or any organoiodine species. Hence it does not change the overall conclusion that kelp is the ultimate cause of the iodine speciation changes we see. Actually sorting out the detailed mechanistic possibilities will require extensive further work outside the scope of this manuscript.

*Macrocystis* forests dominate many shallow temperate to sub-polar rocky reef ecosystems along the continental margins of North and South America, Africa, Australia, New Zealand, and the Southern Ocean Islands [48]. Consequently, *Macrocystis* forests could have significant climatic impacts on a global scale through their processing of iodine, which through fluxes to the atmosphere can result in tropospheric ozone depletion and increased cloud formation, and thus alter patterns of heat retention. However, given the distribution of these forests and in particular the extent of their canopies is highly variable among years, seasons and geographic regions, due to a number of physical and biological forcing factors [46, 47, 56], the influence of these forests is similarly variable, as was observed among seasons in this study.

In summary, several things are immediately clear from our data. First, iodide and iodate concentrations at the Scripps Pier, a location that was outside of the immediate influence of the Point Loma kelp forest, all exhibited very little temporal variation over the course of the study. In contrast, these species concurrently all exhibited high variation within and around the kelp forest. Specifically, as the kelp canopies reached their maximum biomass during the summer, iodide concentrations at the kelp forest increased slightly, reaching their maximum concentrations, while at the same time iodate decreased substantially, reaching their minimum concentrations. Second, while the temporal fluctuations in iodide were negatively related to those of iodate, they were much smaller in magnitude. This suggest that forests are not only impacting iodine speciation, but also that the reduction of iodate from the water column is due to its removal rather than it simply being reduced to iodide. Third, these fluctuations vary depending on location in relation to the kelp forest, with the greatest variation observed within the middle of the forests and the least variation observed 500 m outside the forests. Fourth, the spatial differences in the concentrations of iodide and iodate among locations relative to the kelp forest were smallest during December 2014 through February 2015 (i.e. winter) when the kelp forests were near their minimum biomass and greatest during the summer when the canopies were near their maximum biomass. Lastly, while we did find significant relationships between fluctuations in iodide and both wind speed and ocean temperature, these associations appeared small in comparison to those with kelp canopy biomass. Similarly, we found significant a relationship between iodate and water temperature, but not wind speed, but this again was appeared small in comparison to the relationship with kelp canopy biomass. Together, this suggests a strong role of kelp in the processing of iodine in the coastal ocean relative to other meteorological and/or oceanographic factors. Given the known links between the emission of these iodine species and atmospheric processes such as ozone destruction and cloud formation, our study suggests that spatial and temporal fluctuations in kelp canopy biomass could be important to local and regional climate through the processing of iodine.

## Supporting information

S1 Fig The Point Loma kelp forest. Infrared aerial photo of the Pt. Loma kelp beds in 2014. The white line represents a typical transect.
(DOCX)

S2 Fig Temporal patterns in iodide concentrations within the Point Loma kelp forest. Correlation of iodide concentrations (mean ± standard deviation) as a function of month between August 2014 and July 2015 at five locations within the Pt. Loma kelp forest. The red dotted line represents the mean sea surface temperature, the black dotted line the mean wind speed (offshore) and the black dotted line mean iodide concentration at the Scripps Pier control site.
(DOCX)
