# Correction: The potential role of kelp forests on iodine speciation in coastal seawater

**DOI:** 10.1371/journal.pone.0189559

**Published:** 2017-12-07

**Authors:** Jennifer Gonzales, Teresa Tymon, Frithjof C. Küpper, Matthew S. Edwards, Carl J. Carrano

The following information is missing from the Funding section: Additional funding was provided to FCK by the UK Natural Environment Research Council within the framework of the Oceans 2025 program / WP 4.5.

The images for Figs 2 and 3 are incorrectly switched. The image that appears as Fig 2 should be Fig 3, and the image that appears as Fig 3 should be Fig 2. The figure captions appear in the correct order.

**Fig 2:**
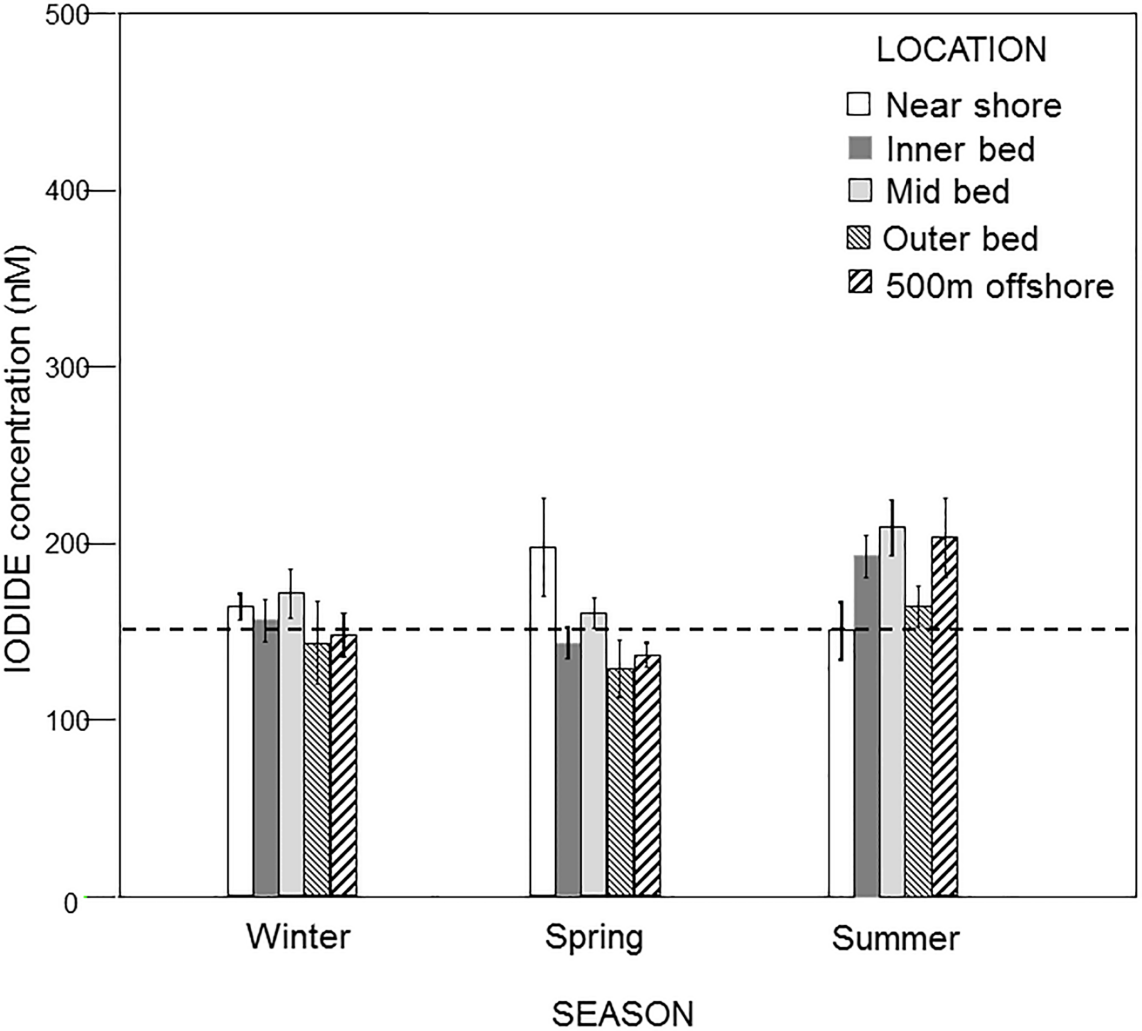
Pooled iodide concentrations (mean ± standard deviation) as a function of season at five locations within the Pt. Loma kelp forest. The dotted line represents the mean iodide concentration at the Scripps Pier control site.

**Fig 3:**
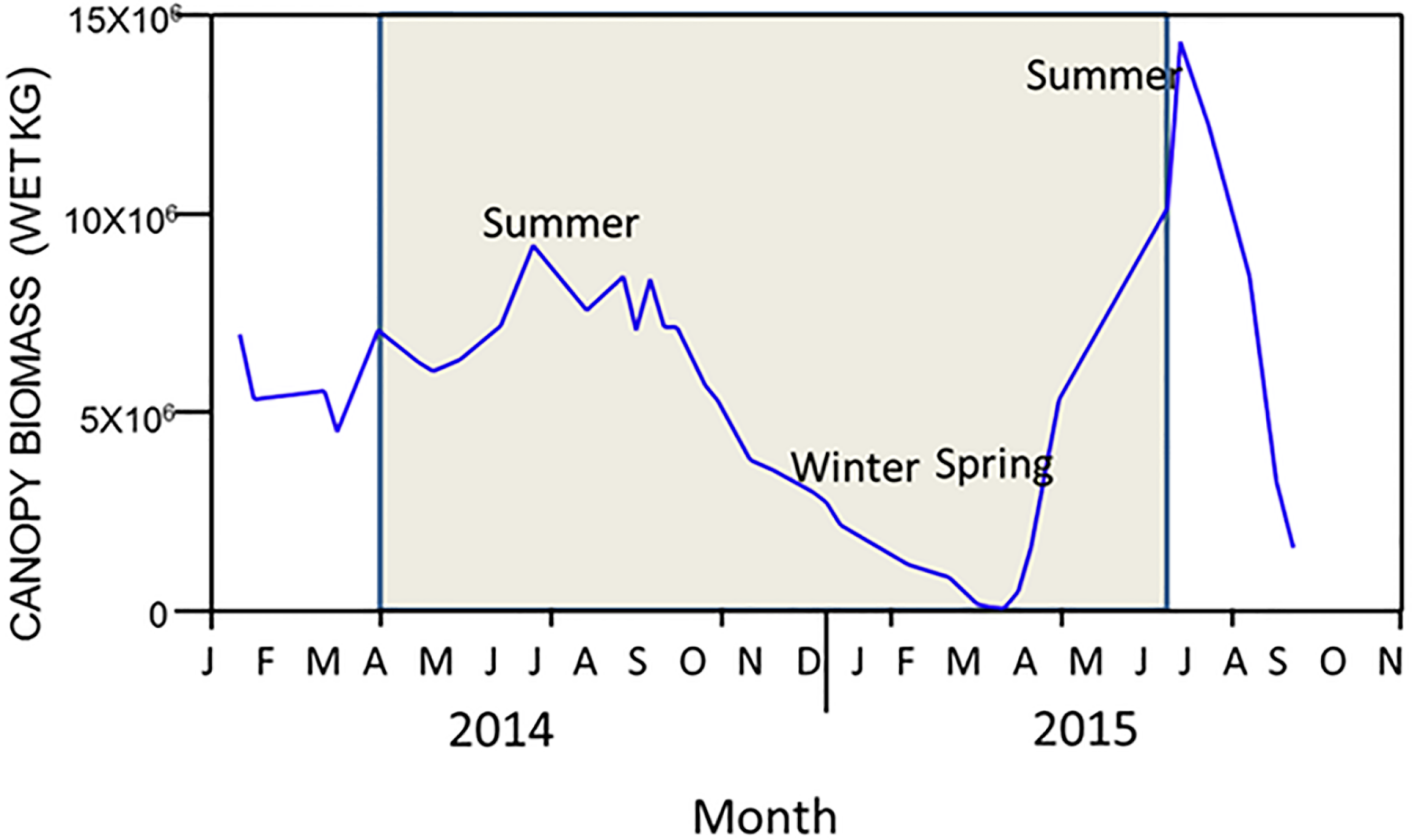
Kelp forest canopy biomass from 2014–2015 as estimated from Landsat imagery, and expressed as biomass in wet kg. The grey box represents the time frame of this study.
