# Supplementary material for: The potential role of kelp forests on iodine speciation in coastal seawater
Source: PLoS One. 2017 Aug 11;12(8):e0180755. doi: 10.1371/journal.pone.0180755 (PMC5553931; doi:10.1371/journal.pone.0180755)
Supplement: S1 Fig — Infrared aerial photo of the Pt. Loma kelp beds in 2014. The white line represents a typical transect. (DOCX) [file pone.0180755.s001.docx]

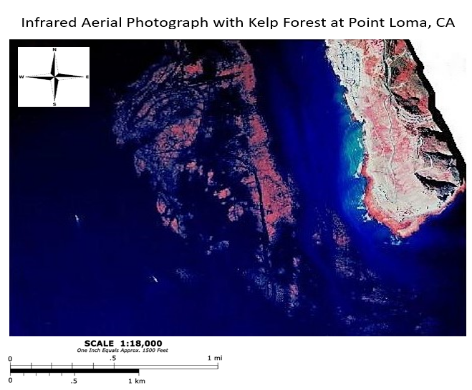


**S1 Fig. The Point Loma Kelp Forest.** Infrared aerial photo of the Pt. Loma kelp beds in 2014. The white line represents a typical transect.
