# Supplementary material for: The potential role of kelp forests on iodine speciation in coastal seawater
Source: PLoS One. 2017 Aug 11;12(8):e0180755. doi: 10.1371/journal.pone.0180755 (PMC5553931; doi:10.1371/journal.pone.0180755)
Supplement: S2 Fig — Correlation of iodide concentrations (mean ± standard deviation) as a function of month between August 2014 and July 2015 at five locations within the Pt. Loma kelp forest. The red dotted line represents the mean sea surface temperature, the black dotted line the mean wind speed (offshore) and the black dotted line mean iodide concentration at the Scripps Pier control site. (DOCX) [file pone.0180755.s002.docx]

**
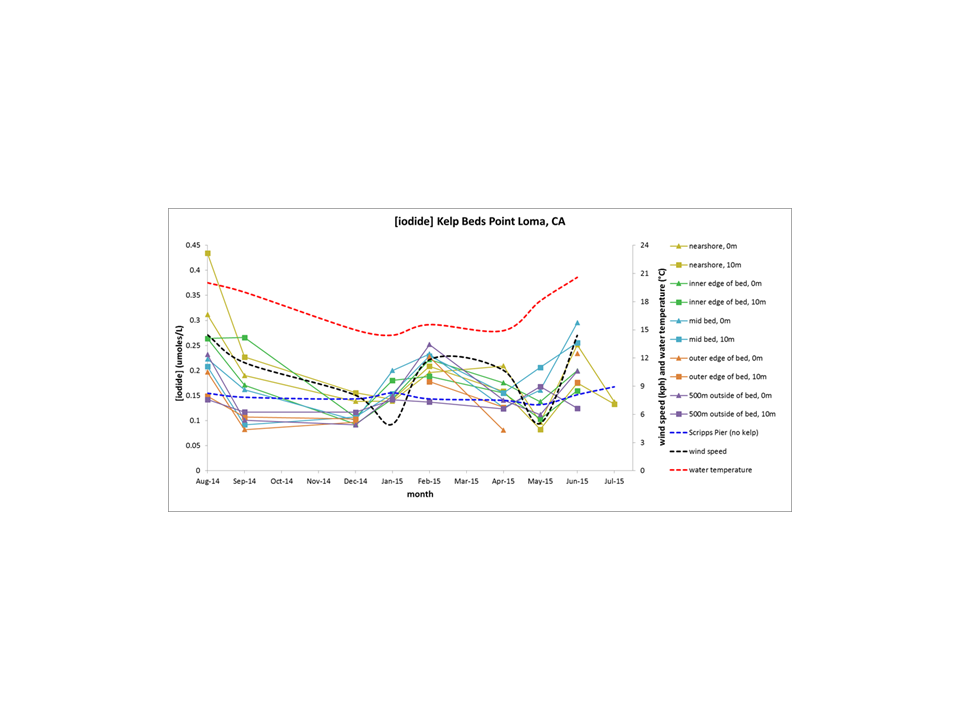
**

**S2 Fig**. **Temporal patterns in Iodide concentrations within the Point Loma Kelp Forest.** Correlation of iodide concentrations (mean ± standard deviation) as a function of month between August 2014 and July 2015 at five locations within the Pt. Loma kelp forest. The red dotted line represents the mean sea surface temperature, the black dotted line the mean wind speed (offshore) and the black dotted line mean iodide concentration at the Scripps Pier control site.
